# Climate change and forest plagues: assessing current and future impacts of diprionid sawflies on the pine forests of north-western Mexico

**DOI:** 10.7717/peerj.7220

**Published:** 2019-07-16

**Authors:** Víctor M. Aguilera-Molina, Khutzy K. Munguía-Ortega, Eulogio López-Reyes, Andrés Martínez-Aquino, F. Sara Ceccarelli

**Affiliations:** 1 Departamento de Biología de la Conservación, Centro de Investigación Científica y de Educación Superior de Ensenada, Ensenada, Baja California, Mexico; 2 Facultad de Ciencias, Universidad Autónoma de Baja California, Ensenada, Baja California, Mexico; 3 Departamento de Biología de la Conservación, CONACYT-Centro de Investigación Científica y de Educación Superior de Ensenada, Ensenada, Baja California, Mexico

**Keywords:** Defoliators, Pests, Climate change, *Pinus jeffreyi*, *Zadiprion*, Diprionidae, Conifer sawfly, Climate modeling, National Park, Baja California

## Abstract

The imminent threat of climate change lies in its potential to disrupt the balance of ecosystems, particularly vulnerable areas such as mountain-top remnant forests. An example of such a fragile ecosystem is the Sierra San Pedro Mártir (SSPM) National Park of Mexico’s Baja California state, where high levels of endemism can be found, and which is home to one of the country’s few populations of the emblematic Jeffrey pine (*Pinus jeffreyi*). Recent outbreaks of pine-feeding sawfly larvae in SSPM increase the vulnerability of this forest ecosystem, calling for immediate assessments of the severity of this threat. Here, we present a thorough study of the sawfly’s biology and distribution, carrying out molecular and morphology-based identification of the species and creating model-based predictions of the species distribution in the area. The sawfly was found to belong to an undescribed species of the genus *Zadiprion* (family Diprionidae) with a one-year life-cycle. The distribution of this species appears to be restricted to the SSPM national park and it will probably persist for at least another 50 years, even considering the effects of climate change.

## Introduction

The health and ecological balance of forests is being compromised by the rising temperatures, CO_2_ concentration and increase in droughts brought about by climate change (Allen et al., 2010; Trenberth et al., 2007). Added to these abiotic stressors, pest species will have stronger adverse effects on weakened trees (Bentz et al., 2010; Haavik et al., 2015; Kolb et al., 2016). For example, the sawfly species of the family Diprionidae (Hymenoptera, Symphyta), also known as conifer sawflies, pose a threat in that the wasp larvae are major defoliators of tree species belonging to genera such as *Pinus* and *Juniperus* (Smith, 1979). With the rising temperatures predicted under the current climate change scenario, pests may also be strengthened. For example, a correlation has been found between higher winter temperatures and the intensity of outbreaks in *Neodiprion sertifer*, one of the most aggressive diprionid defoliator of pines in Europe. This correlation is due to the fact that higher winter temperatures result in a lower mortality rate of *N. sertifer* eggs (Neuvonen, Pekka & Tarmo, 1999).

Diprionidae are restricted to the Northern Hemisphere and to date there are approximately 90 species described worldwide (Goulet & Huber, 1993). The correct identification of many diprionid species remains a challenge, despite the family’s impact on forests, and their biology is generally under-studied. Most diprionids have evolved to be outbreak species, where abrupt and sudden population spikes occur cyclically without a well-defined period (Price, Roininen & Ohgushi, 2005). These outbreaks can take place every 10, 20 or 30 years, and can be hard to predict (González et al., 2014), although life-history traits and external environmental factors play a role in certain species (Larsson, Björkman & Kidd, 1993). Females of the diprionid species *N. sertifer* are thought to be poor fliers (Griffiths, 1959) and have experimentally been found to generally fly for less than a minute at a time, which would mean that they are able to fly between trees spaced out at 20–40 m (Björkman, Larsson & Bommarco, 1997). Thus, sawflies are probably of low vagility and do not tend to disperse large distances. Further studies on behavior, ecology and biology for most diprionid species are scarce.

Even though sawflies remain enigmatic and poorly-studied, outbreaks have been documented in various parts of Mexico. Species belonging to the genera *Zadiprion* and *Neodiprion* of the family Diprionidae have been registered in several states, such as Chihuahua (Olivo, 2011), Durango (Álvarez Zagoya & Díaz Escobedo, 2010; Quiñonez, 2006), Jalisco (Dirección General de Gestión Forestal y de Suelos (DGGFS), 2008), Guerrero and Oaxaca (González et al., 2014). A further notorious case was reported by Smith, Sánchez-Martínez & Ordaz-Silva (2010) in San Luis Potosí, in which *Juniperus flaccida* was attacked by the diprionid *Monoctenus sanchezi*. The particular case reported by Smith, Sánchez-Martínez & Ordaz-Silva (2010) highlights the lack of information on the subject in Mexico, as the species had been erroneously identified until the authors corrected the issue. Not only were the species mis-identified, but mitigation plans or registers of their progress were also missing (Smith, Sánchez-Martínez & Ordaz-Silva, 2010).

As mentioned previously, the correct identification of diprionid species has always been, and remains a challenge. During the past decades, a commonly used tool for complementing morphological-based taxonomic identification in insects has been the molecular-based species “barcoding” (Hajibabaei et al., 2006; Ceccarelli, Sharkey & Zaldívar-Riverón, 2012; Hebert et al., 2016). This technique relies on the sequence identity within species of the mitochondrial cytochrome c oxidase subunit I (COX-1) gene fragment, whereby the genetic sequence of an unknown species is compared to sequences of known species, deposited in online databases such as the National Center for Biotechnology Information (NCBI) and the Barcode of Life Database (BOLD).

Apart from correctly identifying and elucidating the biology of diprionid pest species, predicting their distribution (both present and future) is crucial for setting up mitigation plans. When time and resources are scarce for extensive field surveys, Species Distribution Modeling (SDM) is used to evaluate the probability of a species’ occurrence in a specific geographic area, given a range of predictor variables from its known distribution. Furthermore, SDM can be applied to predict future distributions of species under different climate change scenarios (Peterson et al., 2011). Under the Biotic-Abiotic-Movement model, the predictor variables come from these three sources related to a species’ ecology and biology (Soberón & Peterson, 2005). Traditionally, the abiotic factors (such as temperature and precipitation) determining a species’ niche were the most widely used predictor variables for distribution modeling, due to their relative ease of access. However, the complexity and dynamic nature of species and their ecosystems calls for thorough and careful work on correlative ecological species distribution models, reason for which there have been extensive studies on providing frameworks and techniques for fine-tuning and obtaining more accurate and precise predictions (Anderson, 2013; Holloway, Miller & Gillings, 2016; Guevara et al., 2018; Merow, Smith & Silander, 2013). Just like abiotic factors, biotic interactions are crucial in determining a species’ distributional range (Araújo & Luoto, 2007; Giannini et al., 2013; Wisz et al., 2013) especially in cases where there are close ecological interactions, such as a plant species for specialist herbivores. Additionally, if information on the species’ movement (M)—which includes vagility and/or landscape friction—is available, a more accurate prediction of the distributional range can be obtained (Fischer, Thomas & Beierkuhnlein, 2011; Bouyer et al., 2015; Holloway, Miller & Gillings, 2016).

In the Mexican state of Baja California, the Sierra San Pedro Mártir (SSPM) National Park is of great biological value, since it hosts 16 endemic species of plants, is home to an important community of *Pinus jeffreyi* (Delgadillo-Rodríguez, 1998) and is one of only two areas in Mexico with coniferous forests and shrublands of the Californian floristic province (Minnich et al., 2000). Furthermore, as opposed to areas with the same floral composition in the U.S.A., SSPM is ecologically important because it is not subject to any man-made fire regime (Minnich et al., 2000; Stephens et al., 2007). Historically, the main threats to the trees in the forest of SSPM have been identified as pathogens and bark beetles (Maloney & Rizzo, 2002). However, during the years 2015 and 2016, the first cases of diprionid larvae defoliating *P. jeffreyi* were registered in SSPM (López et al., 2017). Subsequent field surveys have confirmed the presence of this pest species in SSPM. Sierra Juárez, a mountainous area which also hosts *P. jeffreyi*, approximately 160 km north of SSPM was also surveyed, but to date no outbreaks of this kind have been found (Aguilera-Molina, 2016–2017, personal observation).

In this study, the aims are twofold. First, using morphological and molecular techniques of barcoding, we set out to identify the diprionid pest species found in SSPM and to study its life-cycle. Secondly, we create SDMs to infer the probability of this diprionid in Sierra Juárez, as well as its persistence during the following decades in the region under climate change scenarios. We hypothesize that with rising temperatures due to climate change, the diprionid species’ range will be shifted further north.

## Materials and Methods

### Collecting and rearing of sawflies

During the period of September 2016 and October 2017 conifer sawfly larvae outbreaks in SSPM were monitored. To search for sawfly larvae, a 26 km transect was surveyed from the main road, walking the entire transect and visually inspecting all tree species within a 250 m radius from the road. GPS coordinates of the infected trees were recorded. The maximum height of infected trees was found to be three m, meaning that the sawfly larvae could all be collected by hand. Larvae and foliage (branches and leaves the larvae were found feeding on) were collected in buckets which were sealed with a fine mesh. Subsequently, the larvae were taken to the laboratory and placed in sealed plastic containers of 40 × 20 × 25 cm, with the foliage they were found on. The containers remained undisturbed in the laboratory in ambient conditions. During the collections and transportation, no signs of parasitism where noted, although parasitoid diptera did emerge during rearing (see F. S. Ceccarelli et al., 2019, unpublished data). The collecting permit used for this work was authorized by SEMARNAT (SGPA/DGVS/11596/17).

### Morphological and molecular identification

Identifications of the sawflies collected were carried out using morphology of adult males and females using the key provided by Smith, Sánchez-Martínez & Ojeda-Aguilera (2012), and molecular tools. The molecular identification was based on the mitochondrial COX-1 gene fragment. DNA was extracted in Chelex resin from leg tissue of one adult male and one adult female. The COX-1 gene fragment was amplified using the universal primers designed by Folmer et al. (1994) LCOI1490 forward and HCO2198 reverse. Polymerase chain reaction (PCR) mixes contained five uL 5× PCR Buffer (Life Sciences, New York, NY, USA), 10 umol MgCl_2_, 0.25 umol of each dNTP, 0.4 umol of each primer, 0.2 uL Taq polymerase (five U/uL, Life Sciences), two uL genomic DNA and ddH_2_O to bring the final volume to 25 uL. PCR cycling conditions were as follows: 94 °C for 5 min; 35 cycles of 95 °C for 30 s, 48 °C for 30 s, 72 °C for 45 s; 72 °C for 10 min.

The unpurified PCR products were sent to Macrogen Inc., Korea for sequencing in both forward and reverse directions. The sequences were assembled and edited in the program Sequencher v. 4.1.4 (Gene Codes Corporation, Ann Arbor, MI, USA), ensuring there were no termination codons, which would indicate the presence of pseudogenes. The sequences were then compared to the available sequences on online databases BLAST of NCBI (https://blast.ncbi.nlm.nih.gov/Blast.cgi) and the BOLD System barcode project (http://www.boldsystems.org/). The COX-1 sequences of six individuals from the databases with the highest identity scores to the two sequences from this study were downloaded and the eight sequences were aligned in MAFFT v. 7 (Katoh, Rozewicki & Yamada, 2017) using the “Auto” search strategy. Pairwise and net between group mean *p*-distances were then calculated in MEGA7 (Kumar, Stecher & Tamura, 2016) to evaluate the species identity of the sawflies from this study based on evolutionary distances.

### Species distribution models

Since the field sampling was carried out in a spatially non-heterogeneous fashion (i.e., along the sides of a road), probable occurrence points based on the presence of the host plant were extrapolated by creating a grid of coordinates with 0.02 decimal degree increments around each real collection point. A polygon was drawn around the area in SSPM where *P. jeffreyi* is found (Minnich et al., 2000; Franco-Vizcaino et al., 2002) and only the points within the polygon were retained. Spatial thinning was then carried out on the extrapolated data using the spThin package (Aiello-Lammens et al., 2015) for R v. 3.5.3 (R Core Team, 2019) with 100 replicates and a thinning parameter of 0.5 km. The resulting set of points will be referred to as the *extrapolated dataset*, while the dataset containing the original GPS points will be referred to as the *real dataset*.

The predictor variables considered for the species distribution models were 19 bioclimatic variables at 30 arcsecond resolution, downloaded from the WorldClim v. 1.4 database (Hijmans et al., 2005) plus a vegetation raster (downloaded from INEGI https://www.inegi.org.mx/app/areasgeograficas/?ag=02). The vegetation raster was used as a proxy for host plant distribution, since a thorough search for distributional data of the host plant returned unreliable datapoints. Assuming low vagility for the sawfly species (Griffiths, 1959; Björkman, Larsson & Bommarco, 1997), the 20 rasters were cropped to approximately 50 km around the polygon of where the sawfly’s host species, *P. jeffreyi*, is found in SSPM. A greater distance was left in the rasters toward the north of SSPM, to include the area of Sierra Juárez in the analyses, since one of the study’s objectives was to predict the sawfly’s presence in the mountains of Sierra Juárez. Since collinear variables have been shown to hamper results of species distribution models (Dormann et al., 2013), a principal component analysis was carried out on the 20 variables to assess their relative importance and unique contribution in explaining the current known distribution of the diprionid individuals. Of the 20 potential predictor variables, 10 rasters with the highest vector magnitude were selected, and pairwise Pearson’s product-moment correlation tests were run, to select variables that were not positively correlated. Analyses were carried out in R, using the raster package (Hijmans, 2019).

Two potential species distribution models were compared in the R package ENMeval v. 0.3.0 (Muscarella et al., 2014): a model based on the presence data of the *real dataset* vs. a model using the *extrapolated dataset*. Additionally, each dataset was run with the bioclimatic variables selected previously, as well as the full 20 variables (19 bioclimatic and one vegetation). The models were constructed using the maximum entropy algorithm implemented in maxent (Phillips, Anderson & Schapire, 2006) and their suitability was evaluated based on the Akaike information criteria (AIC) scores (Akaike, 1974). To estimate optimal levels of complexity in the models, specific settings were tested with a combination of feature classes (Linear; Quadratic; Linear and Quadratic; Hinge; Linear, Quadratic and Hinge) and regularization multipliers (1.0–5.0 with 0.5 intervals), following Guevara et al. (2018).

The final model with the selected input variables and settings was run under the maxent algorithm in the dismo package v. 1.1-4 (Hijmans et al., 2017) in R, setting aside 25% of the datapoints as random test data and running 500 maximum iterations and a threshold of 0.00001. The predictive model for the present distribution was built using the selected predictor variables. Additionally, the same bioclimatic variables were obtained for future predictions to model potential sawfly distributions in the years 2050 and 2070 under the representative concentration pathways (RCP) 2.6 and 8.5 greenhouse gas scenarios of the NCAR-CCSM4 General Circulation Model, which assume the lowest and highest possible future emissions, respectively. The models were then tested by including the movement component of the species using the R scripts from Holloway, Miller & Gillings (2016). Since the difference between the models produced by the “unlimited” (which is the default for maxent) and “no dispersal” scenarios was negligible, intermediate scenarios were left out.

## Results

**Figure 1:**
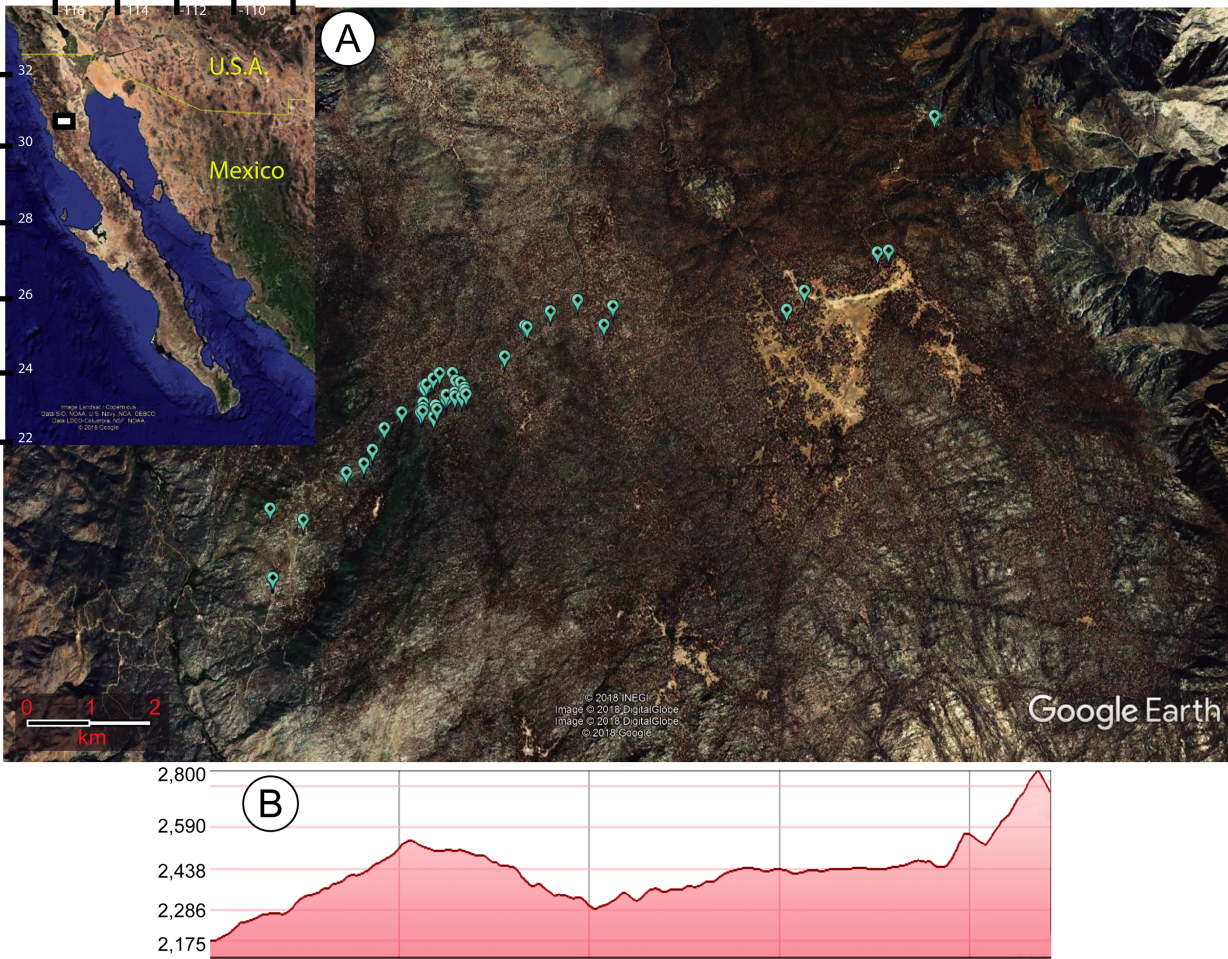
Map of study site. (A) Maps of the area in which sawfly larvae outbreaks were recorded feeding on *Pinus jeffreyi* in the Sierra San Pedro Martir National Park of Mexico’s Baja California state. The inset map shows the study area in white, while the larger map shows the exact points of the infested trees (created in Google Earth with conversion tool from http://www.earthpoint.us/Default.aspx). (B) Elevational profile of the study area, with the altitude shown in meters above sea level.

Within the area surveyed, 47 sawfly larvae-infested individuals of *P. jeffreyi* were found (Fig. 1). Although other trees were inspected for diprionids, *P. jeffreyi* was the only species found infested. A total of 147 larvae from the 47 trees were collected and left to emerge. Of the 147 larvae collected, 21 pupated, of which 19 emerged (ten males, nine females) in August 2018. This means that the emergence in captivity was of 12.9% from larvae and 90.5% from cocoons.

The two sequences of the COX-1 gene fragment obtained in this study had 2/659 variable sites. The search on the BOLD systems database found no immediate match between the queried specimens from this study and the sequences available in the database. The BLAST search retrieved a 95% identity match with a sequence from an individual identified as *Zadiprion rohweri* from the USA (study by Malm & Nyman, 2015; GenBank accession number KF936531). This sequence, and a further COX-1 sequence belonging to *Z. rohweri* (study by Linnen & Farrell (2008); GenBank accession number EU279769) are the only two publicly available COX-1 sequences for the whole genus to date. The pairwise genetic distances between the species in this study and the six species in GenBank with the highest identity are shown in Table 1. The net between group mean distance between the two *Z. rohweri* sequences and the two sequences from this study was 0.0380. The two sequences generated in this study were deposited in GenBank with accession numbers MK940482 and MK940483.

**Table 1:** Estimates of evolutionary divergence between sequences.

		1	2	3	4	5	6	7	8
1	Diprionidae C002								
2	Diprionidae C003	0.0030							
3	*Zadiprion rohweri* KF936531	0.0370	0.0401						
4	*Zadiprion rohweri* EU279769	0.0404	0.0404	0.0000					
5	*Gilpinia hercyniae* GU690155	0.1185	0.1201	0.1156	0.1434				
6	*Gilpinia frutetorum* KC974310	0.1322	0.1307	0.1217	0.1581	0.0973			
7	*Neodiprion abietis* JN294221	0.1459	0.1459	0.1402	0.1324	0.1292	0.1292		
8	*Neodiprion sertifer* KC976255	0.1522	0.1492	0.1451	0.1397	0.1400	0.1279	0.0959	

**Note:**

The number of base differences per site from between sequences are shown. The analysis involved eight nucleotide sequences. Codon positions included were 1st+2nd+3rd+Noncoding. All ambiguous positions were removed for each sequence pair. There were a total of 659 positions in the final dataset. Evolutionary analyses were conducted in MEGA7.

**Figure 2:**
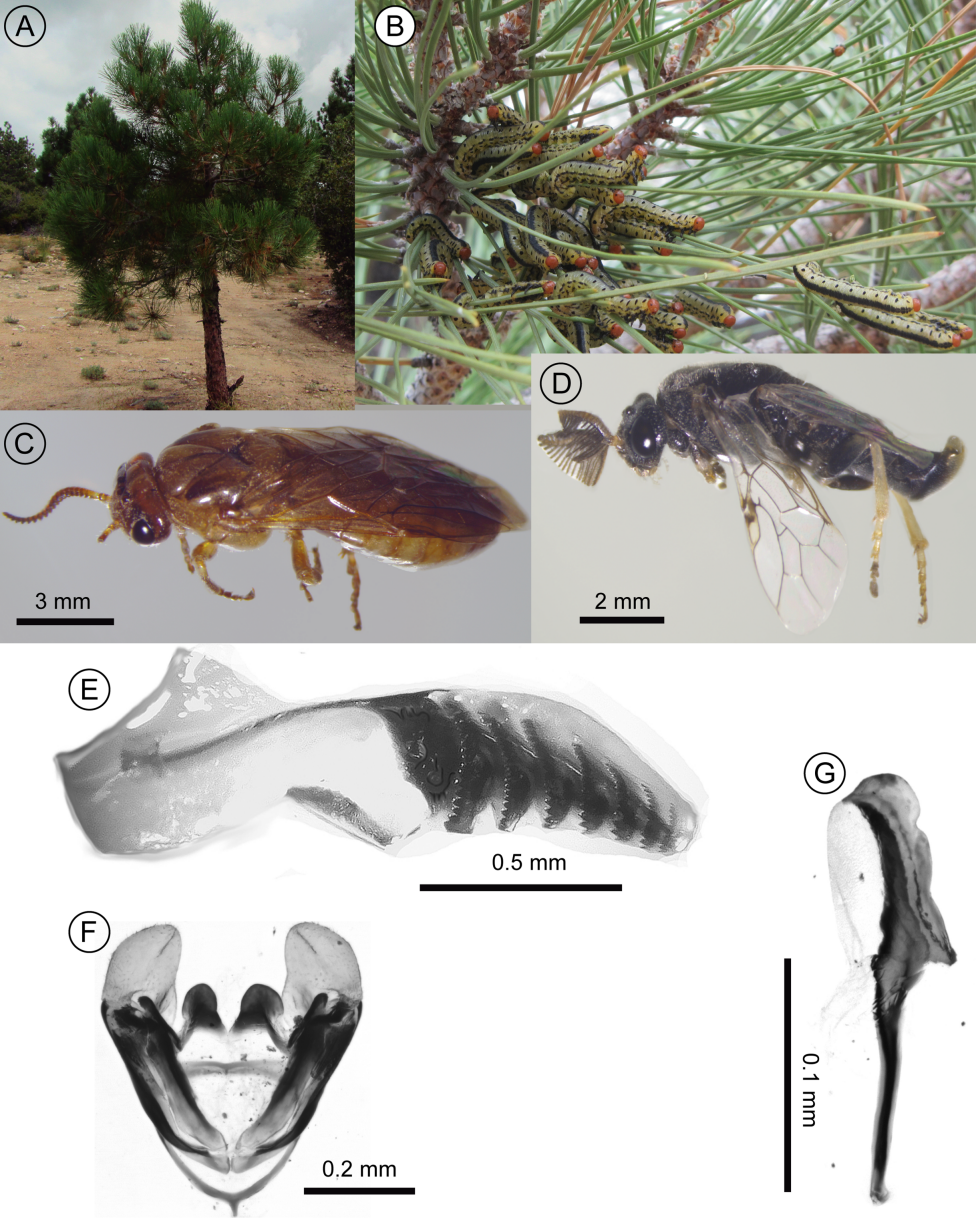
Images of diprionid larvae-infested pine tree, diprionid adults and genitalia. Photographs of (A) a young *Pinus jeffreyi* pine in SSPM infested by *Zadiprion* sp. larvae; (B) close-up of larvae feeding on pine needles; (C) lateral view of female and (D) male adults of *Zadiprion* sp.; (E) female lancet, (F) male genital capsule and (G) lateral view of male penis valve. Photographs by V.M. Aguilera-Molina (A, B), K. Munguia-Ortega (C, D) and F.S. Ceccarelli (E–G).

Based on morphology, the specimens in this study were confirmed to belong to the genus *Zadiprion*. However, the key and descriptions provided by Smith, Sánchez-Martínez & Ojeda-Aguilera (2012) for the six species described from Mexico (and the southern USA) did not match with the morphological characters of the specimens here (see Fig. 2). Based on the key, the specimens here fall most closely with *Z. rohweri* in that the first annulus of the female’s lancet is present and has several teeth on an inverted *U*-shaped pattern near the dorsal margin and the mesepisternum is entirely yellow. The difference between *Z. rohweri* and the specimens in this study is that in the latter, the distal end of the lancet is more rounded and has one annulus less. In the males of the species, the specimens here lack *Z. rohweri*’s distinctive “yellow spot near the upper inner orbit of each eye,” and while the description male’s penis valve and genital capsule generally coincide, the apical structures in *Z. rohweri* are less rounded, the overall proportions are narrower (see figures in Smith, Sánchez-Martínez & Ojeda-Aguilera, 2012 and Figs. 2E–2G).

The species distribution models were run with the *real dataset*, applying linear, quadratic and hinge features, a regularization multiplier of 5, and five bioclimatic variables, based on the lowest AIC score. The five bioclimatic variables retained for the models were annual mean temperature, annual precipitation, mean diurnal range, maximum temperature of warmest month and minimum temperature of coldest month. Of these, the annual mean temperature is the variable which best predicts the distribution of the *Zadiprion* species found in SSPM.

**Figure 3:**
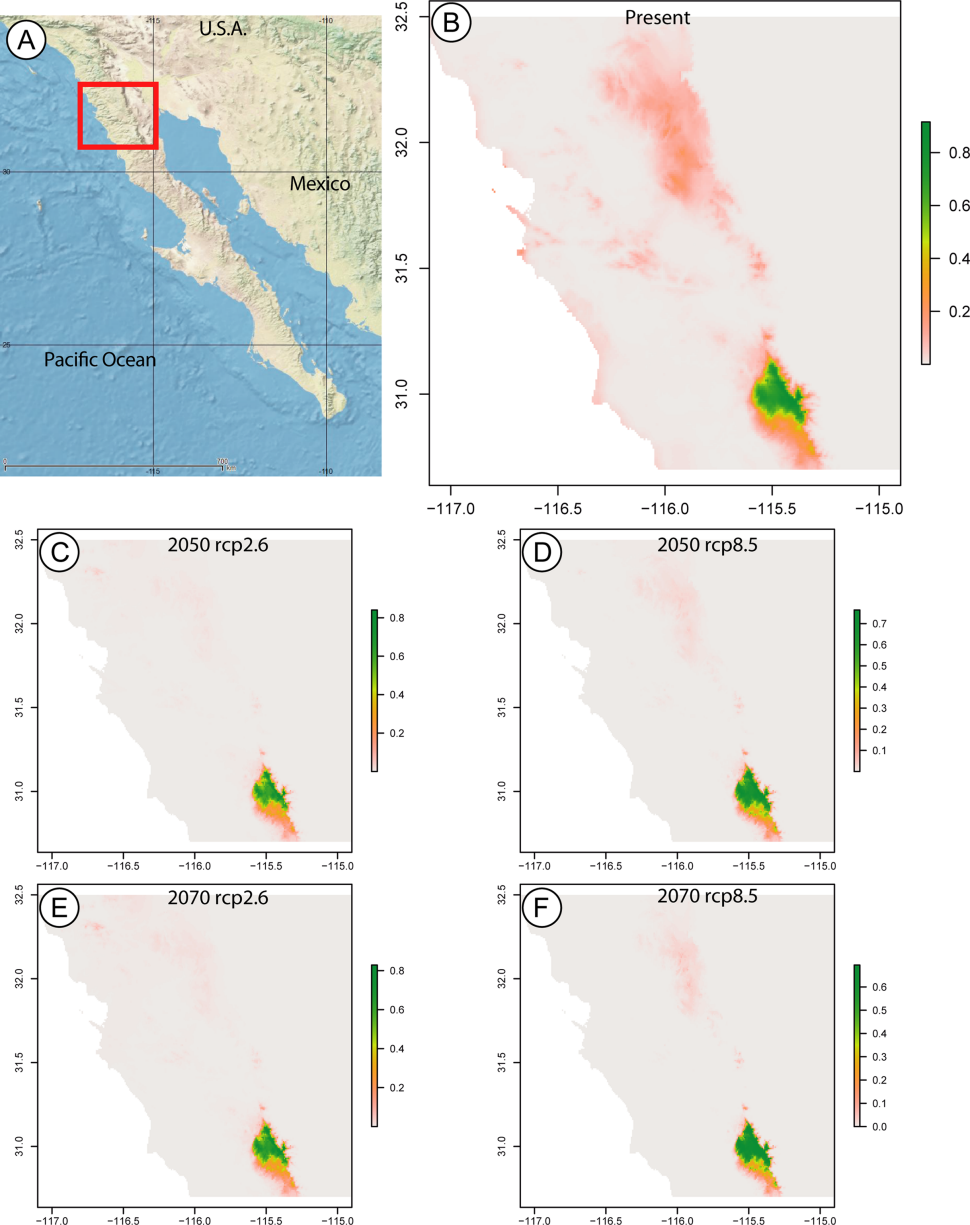
Species distribution maps based on climatic niche modeling. (A) Map of the Baja California peninsula showing the area delimited for the species distribution models, represented by a red rectangle. The remaining maps, produced by Maximum Entropy Modeling in maxent, show the probable distribution of the *Zadiprion* species from this study based on bioclimatic niche modeling. Bioclimatic based predictions are shown for (B) present-day distribution, as well as future years (C) 2050 assuming lower (RCP2.6), (D) 2050 assuming higher (RCP8.5), (E) 2070 assuming lower (RCP2.6) and (F) 2070 assuming higher (RCP8.5) greenhouse gas emissions. The color scale bar represents the probability of the species presence in a given area.

The maxent model predicting the current distributional range of this species returned a map with a probability greater than 0.8 for a range limited to SSPM, and a probability below 0.3 of the species being found in Sierra Juárez. In the years 2050 and 2070, considering low (RCP2.6) and high (RCP8.5) greenhouse gas emissions, the species is still only predicted to occur in SSPM, with slightly larger areas at probabilities above 0.8 under the RCP8.5 scenario for both years (Fig. 3).

## Discussion

The approximately 13% emergence under laboratory conditions of adults from larvae collected in this study is difficult to assess and compare to other cases, since diprionid sawflies are known to follow patterns of periodic outbreaks which can vary greatly in abundance and density from one year to the next and as they can spontaneously appear, they can also disappear after a number of years (Hanski, 1987). A few studies have looked at diprionid emergence rate, however, mostly in relation to the different types of parasitoids that can affect sawflies. For example, between 80% and 97% of cocoons of *Microdiprion pallipes* emerged as adults in a study by Olofsson (1994), which is comparable to the number found in this study (90.5%), although here we did not investigate the exact causes of pupa mortality. Larval survival in diprionids was also found to correlate with foliar quality of the host plant in *Neodiprion abietis* (Moreau et al., 2003).

Regarding the identification of the diprionid in this study, the evolutionary divergence based on pairwise *p*-distances in the COX-1 gene fragment with *Z. rohweri* suggests that the individuals in this study most likely belong to the genus *Zadiprion*. However, a species-level identification cannot be obtained confidently, since a genetic distance greater than 2% for COX-1 indicates species-level divergence in most diprionids (Schmidt et al., 2017), even though for proper species delimitation, a percent divergence threshold is unreliable and more in-depth analyses with larger sample sizes need to be carried out. In this study we are most likely dealing with an undescribed species, awaiting formal taxonomic description, which will be carried out in the near future, as alpha taxonomy is beyond the scope of this study and will require more individuals to be examined.

The genus *Zadiprion* has been documented on several occasions from Mexico, with six species described to date (Smith, Sánchez-Martínez & Ojeda-Aguilera, 2012). The southern Mexican species *Z. howdeni* has only been recorded on *P. oaxacana*, while in central Mexico, *Z. falsus* (from Jalisco and Michoacán) is found on several species belonging to the genus *Pinus* and for *Z. roteus* (from Hidalgo) the host plant is unknown. In the northern Mexican state of Chihuahua, there are two species, the endemic *Z. ojedae* which mainly feeds on *P. durangensis* but occasionally also on *P. arizonica* and *P. herrerai*, and *Z. townsendi*, which feeds on *P. ponderosa* and is also found in the southern USA. Finally, *Z. rohweri* has been recorded from the southern USA on *P. monophylla* and *P. edulus* (Smith, Sánchez-Martínez & Ojeda-Aguilera, 2012) and in Mexico’s northern state of Coahuila feeding on *P. cembroides* (Smith et al., 2016).

The predicted potential range of the *Zadiprion* species found here, based on maximum entropy modeling, resembles the range of a microendemic species. The issues with geographic scale and the often arbitrary nature of defining areas of endemism have been pointed out repeatedly (Anderson, 1994; Peterson & Watson, 1998), but if we take the concept of endemism to apply to a species restricted to a geographic range, such as the Baja California Mountain Range, then a species occurring only on a single mountain, such as SSPM, could be considered microendemic. While this phenomenon of microendemism may hold true, considering the uniqueness and high level of endemism of other biota in SSPM (Minnich et al., 1997; Riemann & Exequiel, 2007), it may also be an artifact of incomplete sampling of the *Zadiprion* species. Further investigation is required to ascertain whether this sawfly species is found in other areas, or whether it is in fact a SSPM endemic, which resides there in very low numbers until it cyclically undergoes major outbreaks. Another important aspect to investigate is the evolution and historical biogeography of this particular *Zadiprion* species. However, to study its evolution we would require more extensive sampling of closely related species to at least identify its sister species (presumably *Z. rohweri*) through molecular phylogenetic analyses. While molecular phylogenetic studies with macroevolutionary and biogeographical implications have been carried out on the genus *Neodiprion* (Linnen & Farrell, 2008), a robust phylogeny of *Zadiprion* is still lacking.

Considering the fact that the outbreak studied here is the first of its kind formally recorded for SSPM, it is important to be able to predict future outbreaks that could aggravate the fate of *P. jeffreyi* in this area. The new *Zadiprion* species appears to be a specialist feeder on *P. jeffreyi* and as such, this biotic interaction must be taken into account when making model-based predictions of current and future distributions. The fact that the vegetation raster was not among the strongest predictor variables is probably due to the raster’s lack of resolution, since the distribution of this sawfly species is likely to be directly determined by the presence of *P. jeffreyi*. Based on the maxent predictions from this study, it is probable that the sawfly will persist in this area until at least 2070. Beyond this date, it is likely that due to climate change, *P. jeffreyi* will disappear from SSPM at some point, and with continued defoliator outbreaks weakening populations of this pine tree, both defoliator and tree populations could disappear from SSPM in the foreseeable future.

## Conclusions

In this study, we provide a more detailed picture of the biology and distribution of a newly-registered sawfly species which represents an emergent threat to a vulnerable ecosystem: the pine forests of north-western Mexico’s SSPM national park. The sawflies were identified as a new species belonging to the genus *Zadiprion* with a yearly life-cycle and an emergence rate comparable to other sawfly species. Predictions made with climate-based models are that the species is likely to persist in SSPM during the next 50 years, but contrary to our hypothesis, the probability of their range shifting toward the north during the next 50 years is extremely low. To make more accurate predictions about both *P. jeffreyi* and *Zadiprion*, it would be greatly beneficial to carry out a long-term monitoring study while also working with genetic data, which could give better insights into the spatio-temporal movements of the populations and their genetic structure.

## Supplemental Information

10.7717/peerj.7220/supp-1 Supplemental Information 1 Raw data of GPS points where diprionid larvae were found.

10.7717/peerj.7220/supp-2 Supplemental Information 2 DNA sequences. DNA sequences of COX-1 gene fragment of adult sawflies.

## Additional Information and Declarations

### Field Study Permissions

The following information was supplied relating to field study approvals (i.e., approving body and any reference numbers): The collecting permit used for this work was authorized by the Secretaría de Medio Ambiente y Recursos Naturales (SEMARNAT), Mexico (SGPA/DGVS/11596/17).

### DNA Deposition

The following information was supplied regarding the deposition of DNA sequences: The sequences are available in GenBank: MK940482 and MK940483.

### Data Availability

The following information was supplied regarding data availability: The raw data used for the climatic modeling is available in the Supplemental File.
